# Incidental findings in the oncology patient: ovaries

**DOI:** 10.1186/1470-7330-14-S1-O46

**Published:** 2014-10-09

**Authors:** Rosemarie Forstner

**Affiliations:** 1 Department of Radiology, University of Salzburg, Salzburg, Austria

## 

Ovarian incidentalomas are reported in 5-18% of asymptomatic females in cross sectional imaging. Even in oncologic patients the majority of these lesions will be benign, with hydrosalpinx and ovarian cysts as leading diagnoses. Special emphasis should be given to adnexal lesions in patients with history of primaries from the GI tract or breast cancer, both of which have a propensity to metastasize to the ovaries. Patients with hereditary cancer syndromes, e. g BRCA1 or 2, are at a higher risk to develop ovarian cancer. Guidelines for the management of adnexal incidentalomas have recently been developed. Multiparametric MRI aids in further characterization of a complex adnexal lesion and thus assists in patient management in a multidisciplinary setting.

